# *In vitro* treatment of human granulosa cells with irisin and leptin: Quantitative RT-PCR array data (female infertility panel)

**DOI:** 10.1016/j.dib.2021.107781

**Published:** 2022-01-01

**Authors:** Radoslav Stojchevski, Tomer Singer, Karina Ziskovich, Leonid Poretsky, Dimiter Avtanski

**Affiliations:** a Friedman Diabetes Institute, Northwell Health, New York, NY, USA; b Shady Grove Fertility Clinic, New York, NY, USA; c Feinstein Institutes for Medical Research, Northwell Health, Manhasset, NY, USA; d Donald and Barbara Zucker School of Medicine at Hofstra/Northwell, Hempstead, NY, USA

**Keywords:** PCR array, Ovary, Granulosa cells, Irisin, Leptin, Infertility

## Abstract

Reproduction is closely related to energy metabolism: physical activity and adiposity (either insufficient weight or obesity) can affect female fertility. Irisin is a myo- and adipokine produced by skeletal muscles during exercise or shivering as well as in smaller amounts by subcutaneous visceral adipocytes [1]. Leptin is a neuroendocrine adipokine regulating satiety and energy expenditure. Circulating levels of both, irisin and leptin, correlate with adiposity status and physical activity [2], [3], [4], [5], [6]. This article presents data from quantitative PCR array of the *in vitro* effects of irisin and leptin on cultured human ovarian granulosa cells. Granulosa cells were purified from follicular fluid samples obtained from women undergoing *in vitro* fertilization (IVF) procedure and were subjected to treatment with irisin (500 ng/mL) or leptin (100 ng/mL) for 24 h. The array included 84 genes involved in female fertility.

## Specifications Table

**Table:** 

Subject	Health and medical sciences
Specific subject area	Female reproductive endocrinology
Type of data	Table Figure
How the data were acquired	Quantitative RT-PCR array using RT^2^ Profiler PCR Array – Human Female Infertility kit (Qiagen) and QuantStudio 3 Real-Time PCR system (Thermo Fisher Scientific). Data were analyzed using GeneGlobe Data Analysis Center (Qiagen).
Data format	Raw and Analyzed
Description of data collection	Human ovarian granulosa cells were purified from follicular fluid samples obtained during IVF procedure. Cells were cultured *in vitro* and were subjected to treatment with irisin (500 ng/mL) or leptin (100 ng/mL) for 24 h. The effect of irisin and leptin on modulation of 84 genes involved in female infertility was evaluated by using qRT-PCR array.
Data source location	• Institution: Friedman Diabetes Institute, Northwell Health • City/Town/Region: New York, NY • Country: USA
Data accessibility	Repository name: Mendeley Data Data identification number: 10.17632/gr3dg36nzx.1 Direct URL to data: https://data.mendeley.com/datasets/gr3dg36nzx/1

## Value of the Data

• Provided data are useful for understanding the relationship between energy metabolism and reproduction.
• Presented data may be of interest to researchers and physicians working in the field of reproduction.
• This dataset can be used as a basis for further investigations of the molecular mechanisms of irisin and leptin action in the ovary.

## 1 Data Description

**Fig. 1:**
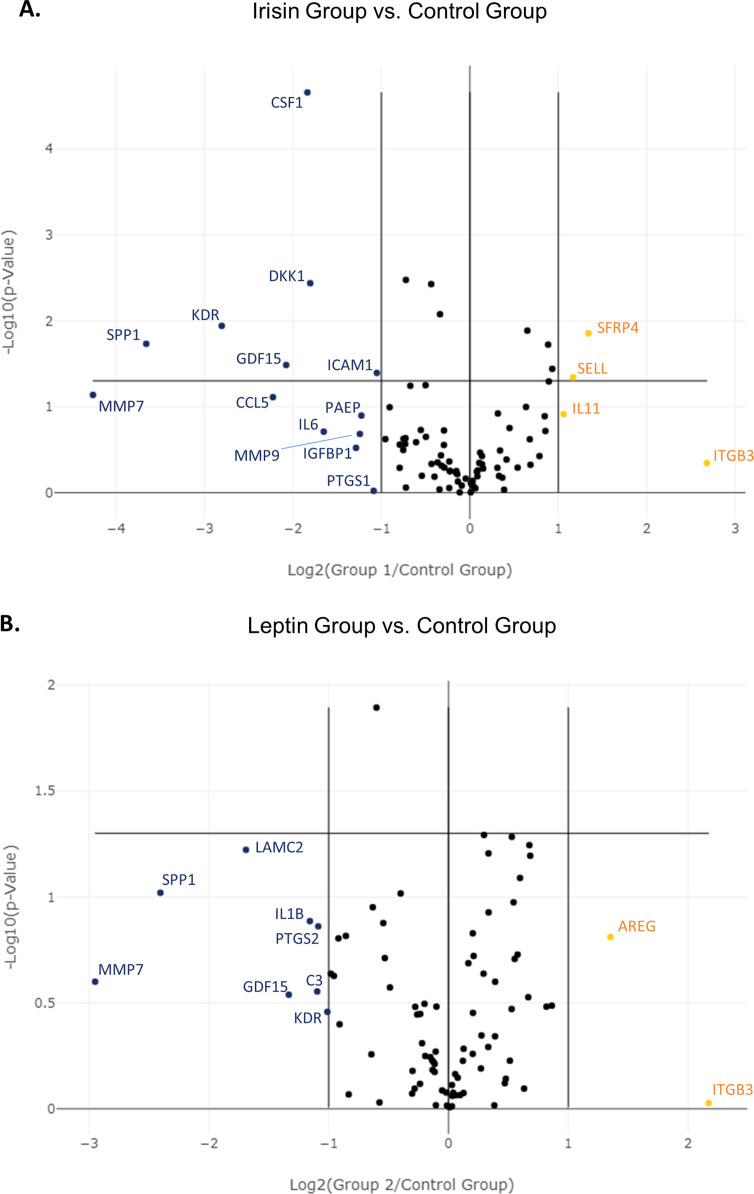
**Volcano plot visualization of the effect of irisin (А) and leptin (B) on the expression of genes related to female infertility.** Volcano plot graph presenting genes upregulated or downregulated more than two-fold. Genes upregulated more than two-fold (in orange) by the effect of irisin (A) are: IL11, SELL, SFRP4 and ITGB3, while genes downregulated more than two-fold (in blue) by the effect of irisin (A) are: ICAM1, PTGS1, PAEP, MMP9, IGFBP1, IL6, DKK1, CSF1, GDF15, CCL5, KDR, SPP1 and MMP7. Genes upregulated more than two-fold (in orange) by the effect of leptin (B) are AREG and ITGB3, while genes downregulated more than two-fold (in blue) by the effect of leptin (B) are: KDR, C3, PTGS2, IL1B, GDF15, LAMC2, SPP1 and MMP7.

**Fig. 2:**
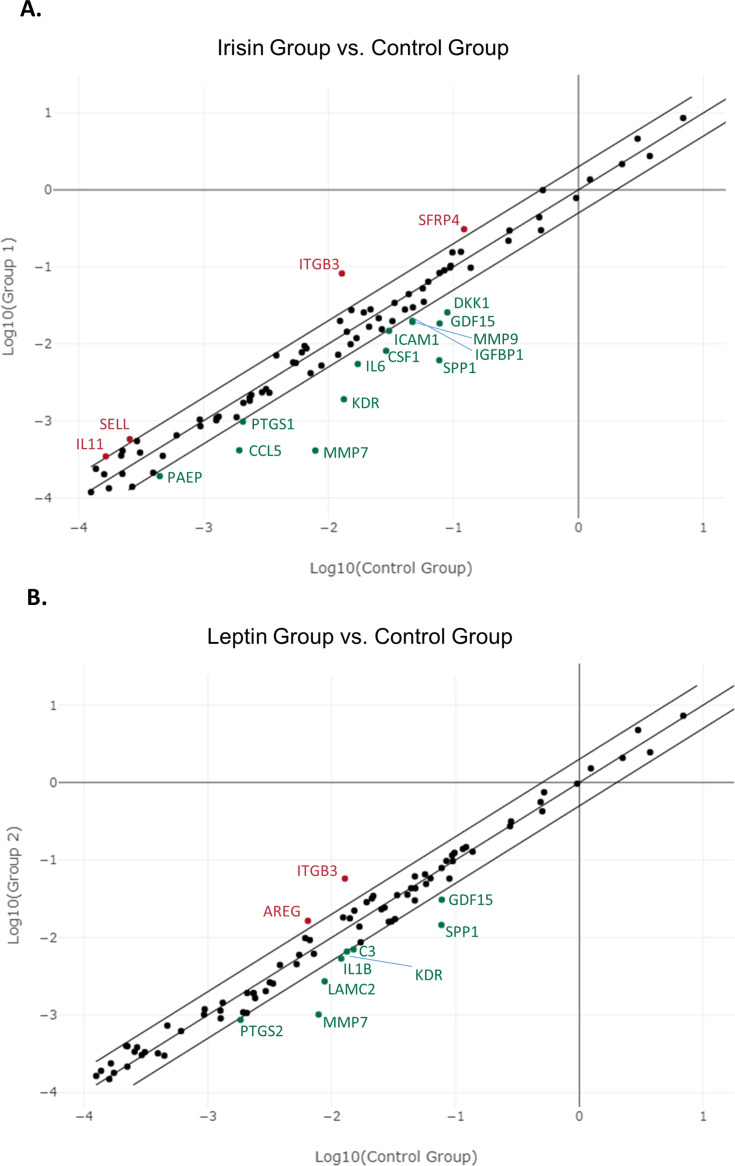
**Scatter plot visualization of the effect of irisin (A) and leptin (B) on the expression of genes related to female infertility.** Scatter plot graph presenting genes upregulated or downregulated more than two-fold. Genes upregulated more than two-fold (in red) by the effect of irisin (A) are: IL11, SELL, SFRP4 and ITGB3, while genes downregulated more than two-fold (in green) by the effect of irisin (A) are: ICAM1, PTGS1, PAEP, MMP9, IGFBP1, IL6, DKK1, CSF1, GDF15, CCL5, KDR, SPP1 and MMP7. Genes upregulated more than two-fold (in red) by the effect of leptin (B) are AREG and ITGB3, while genes downregulated more than two-fold (in green) by the effect of leptin (B) are: KDR, C3, PTGS2, IL1B, GDF15, LAMC2, SPP1 and MMP7.

This article presents data obtained from qRT-PCR array measuring the expression of various genes related to female infertility. Human ovarian granulosa cells purified from follicular fluid samples were grown *in vitro* and treated with irisin or leptin for 24 h. Array included 84 genes listed in Tables 1 and 2. Raw data from the analysis (C*t* values) have been published in a data repository [7]. Analyzed data demonstrating fold-regulation and fold-change of irisin or leptin treatment *vs.* control are presented in Tables 3 and 4, Figs. 1 and 2.

**Table 1:** List of genes included in the PCR array.

Gene Symbol	Gene full name	UniGene ID	GeneBank ID
AKT1	V-Akt murine thymoma viral oncogene homolog 1	Hs.525622	NM_005163
ANXA2	Annexin A2	Hs.511605	NM_004039
APOD	Apolipoprotein D	Hs.522555	NM_001647
AR	Androgen receptor	Hs.496240	NM_000044
AREG	Amphiregulin	Hs.270833	NM_001657
BAX	BCL2-associated X protein	Hs.624291	NM_004324
BCL2	B-cell CLL/lymphoma 2	Hs.150749	NM_000633
C2	Complement component 2	Hs.408903	NM_000063
C3	Complement component 3	Hs.529053	NM_000064
CALCA	Calcitonin-related polypeptide, alpha	Hs.37058	NM_001741
CASP3	Caspase 3	Hs.141125	NM_004346
CCL5	Chemokine (C—C motif) ligand 5	Hs.514821	NM_002985
CCNB1	Cyclin B1	Hs.23960	NM_031966
CD55	CD55 molecule	Hs.126517	NM_000574
CDH1	Cadherin 1	Hs.461086	NM_004360
CFD	Complement factor D	Hs.155597	NM_001928
CLDN4	Claudin 4	Hs.729359	NM_001305
COMP	Cartilage oligomeric matrix protein	Hs.1584	NM_000095
CRABP2	Cellular retinoic acid binding protein 2	Hs.405662	NM_001878
CSF1	Colony stimulating factor 1	Hs.591402	NM_000757
CTNNB1	Catenin, beta 1	Hs.476018	NM_001904
CXCL12	Chemokine (C-X-C motif) ligand 12	Hs.522891	NM_000609
DKK1	Dickkopf homolog 1	Hs.40499	NM_012242
EGF	Epidermal growth factor	Hs.419815	NM_001963
EGFR	Epidermal growth factor receptor	Hs.488293	NM_005228
ESR1	Estrogen receptor 1	Hs.208124	NM_000125
ESR2	Estrogen receptor 2	Hs.729020	NM_001437
F3	Coagulation factor III	Hs.62192	NM_001993
FBN1	Fibrillin 1	Hs.591133	NM_000138
FN1	Fibronectin 1	Hs.203717	NM_002026
GADD45A	Growth arrest and DNA-damage-inducible, alpha	Hs.80409	NM_001924
GAST	Gastrin	Hs.2681	NM_000805
GDF15	Growth differentiation factor 15	Hs.616962	NM_004864
GPX3	Glutathione peroxidase 3	Hs.386793	NM_002084
HBEGF	Heparin-binding EGF-like growth factor	Hs.799	NM_001945
HOXA10	Homeobox A10	Hs.110637	NM_018951
HOXA11	Homeobox A11	Hs.249171	NM_005523
ICAM1	Intercellular adhesion molecule 1	Hs.643447	NM_000201
IGF1	Insulin-like growth factor 1	Hs.160562	NM_000618
IGFBP1	Insulin-like growth factor binding protein 1	Hs.642938	NM_000596
IL11	Interleukin 11	Hs.467304	NM_000641
IL15	Interleukin 15	Hs.654378	NM_000585
IL1A	Interleukin 1, alpha	Hs.1722	NM_000575
IL1B	Interleukin 1, beta	Hs.126256	NM_000576
IL1R1	Interleukin 1 receptor, type I	Hs.701982	NM_000877
IL6	Interleukin 6	Hs.654458	NM_000600
ITGA4	Integrin, alpha 4	Hs.694732	NM_000885
ITGAV	Integrin, alpha V	Hs.436873	NM_002210
ITGB3	Integrin, beta 3	Hs.218040	NM_000212
KDR	Kinase insert domain receptor	Hs.479756	NM_002253
LAMC2	Laminin, gamma 2	Hs.591484	NM_005562
LEP	Leptin	Hs.194236	NM_000230
LIF	Leukemia inhibitory factor	Hs.2250	NM_002309
LIFR	Leukemia inhibitory factor receptor, alpha	Hs.133421	NM_002310
MAOA	Monoamine oxidase A	Hs.183109	NM_000240
MID1	Midline 1	Hs.27695	NM_000381
MKI67	Antigen identified by monoclonal antibody Ki-67	Hs.689823	NM_002417
MMP2	Matrix metallopeptidase 2	Hs.513617	NM_004530
MMP7	Matrix metallopeptidase 7	Hs.2256	NM_002423
MMP9	Matrix metallopeptidase 9	Hs.297413	NM_004994
MSX1	Msh homeobox 1	Hs.424414	NM_002448
MUC1	Mucin 1	Hs.89603	NM_001018016
OLFM1	Olfactomedin 1	Hs.522484	NM_006334
PAEP	Progestagen-associated endometrial protein	Hs.532325	NM_002571
PCNA	Proliferating cell nuclear antigen	Hs.728886	NM_182,649
PGF	Placental growth factor	Hs.252820	NM_002632
PGR	Progesterone receptor	Hs.32405	NM_000926
PRL	Prolactin	Hs.1905	NM_000948
PTGS1	Prostaglandin-endoperoxide synthase 1	Hs.201978	NM_000962
PTGS2	Prostaglandin-endoperoxide synthase 2	Hs.196384	NM_000963
SELL	Selectin L	Hs.728756	NM_000655
SFRP4	Secreted frizzled-related protein 4	Hs.658169	NM_003014
SOD1	Superoxide dismutase 1	Hs.443914	NM_000454
SPP1	Secreted phosphoprotein 1	Hs.313	NM_000582
STAT3	Signal transducer and activator of transcription 3	Hs.463059	NM_003150
STMN1	Stathmin 1	Hs.209983	NM_005563
TGFB1	Transforming growth factor, beta 1	Hs.645227	NM_000660
TIMP1	TIMP metallopeptidase inhibitor 1	Hs.522632	NM_003254
TNF	Tumor necrosis factor	Hs.241570	NM_000594
TNFRSF10B	Tumor necrosis factor receptor superfamily, member 10b	Hs.521456	NM_003842
TP53	Tumor protein p53	Hs.654481	NM_000546
VCAM1	Vascular cell adhesion molecule 1	Hs.109225	NM_001078
VEGFA	Vascular endothelial growth factor A	Hs.73793	NM_003376
WNT2	Wingless-type MMTV integration site family, member 2	Hs.567356	NM_003391
ACTB	Actin, beta	Hs.520640	NM_001101
B2M	Beta-2-microglobulin	Hs.534255	NM_004048
GAPDH	Glyceraldehyde-3-phosphate dehydrogenase	Hs.592355	NM_002046
HPRT1	Hypoxanthine phosphoribosyltransferase 1	Hs.412707	NM_000194
RPLP0	Ribosomal protein, large, P0	Hs.546285	NM_001002

**Table 2:** PCR array genes organized by function.

Function	Gene
Disregulated during infertility	CFD, CLDN4, COMP, CRABP2, DKK1, ESR2, GADD45A, GAST, GDF15, GPX3, IGFBP1, IL15, MAOA, MSX1, OLFM1, PAEP, SFRP4, SPP1
Receptive endometrium	AREG, CALCA, CSF1, EGF, HOXA10, HOXA11, LEP, LIF, LIFR, MUC1, PAEP, PGR, PTGS1, PTGS2, SELL
Signal transduction	
IL-1 signaling	IL1A, IL1B, IL1R1
Wnt signaling	CDH1, CTNNB1, DKK1, MMP7, PTGS2, SFRP4, TP53, WNT2
Prostaglandin signaling	PTGS1, PTGS2
Other signal transduction genes	AKT1, EGF, EGFR, GDF15, STAT3, TGFB1
Leukocyte migration	CALCA, CCL5, CLDN4, CTNNB1, CXCL12, FBN1, FN1, ICAM1, ITGA4, ITGAV, ITGB3, LAMC2, MMP2, MMP7, MMP9, SELL, SPP1, VCAM1
Cell cycle	CCNB1, GADD45A, MKI67, PCNA, STMN1, TGFB1, TP53
Coagulation	C2, C3, CD55, CFD, F3
Apoptosis	BAX, BCL2, CASP3, COMP, GADD45A, IL1B, MSX1, TNF, TNFRSF10B, TP53
Cytokines	CCL5, CSF1, CXCL12, IL11, IL15, IL1A, IL1B, IL1R1, IL6, LIFR, TGFB1
Other genes involved in pregnancy	ANXA2, APOD, AR, ESR1, HBEGF, IGF1, KDR, MID1, PGF, PRL, SOD1, TIMP1, VEGFA

**Table 3:** Effect of irisin on the expression of genes related to female infertility.

Gene Symbol	Fold Regulation	Fold Change	p-Value	Significance
ANXA2	-1.23	0.82	0.189	ns
APOD	-1.35	0.74	0.4628	ns
AR	1.03	1.03	0.8525	ns
AREG	1.45	1.45	0.5115	ns
BAX	1.02	1.02	0.806	ns
BCL2	-1.17	0.86	0.5586	ns
C2	-1.18	0.85	0.8827	ns
C3	-1.52	0.66	0.2588	ns
CALCA	-1.07	0.94	0.8268	ns
CASP3	-1.18	0.85	0.434	ns
CCL5	-4.68	0.21	0.0774	ns
CCNB1	1.01	1.01	0.7758	ns
CD55	1.06	1.06	0.5712	ns
CDH1	-1.94	0.52	0.2382	ns
CFD	1.11	1.11	0.5235	ns
CLDN4	-1.17	0.85	0.5629	ns
COMP	1.26	1.26	0.6391	ns
CRABP2	-1.73	0.58	0.276	ns
CSF1	-**3.57**	**0.28**	**0.000022**	****
CTNNB1	1.06	1.06	0.5502	ns
CXCL12	1.06	1.06	0.6446	ns
DKK1	-**3.49**	**0.29**	**0.0037**	**
EGF	-1.11	0.9	0.5601	ns
EGFR	-1.42	0.71	0.0562	ns
ESR1	-1.25	0.8	0.485	ns
ESR2	1.31	1.31	0.9232	ns
F3	-1.1	0.91	0.6093	ns
FBN1	1.04	1.04	0.8893	ns
FN1	1.55	1.55	0.1007	ns
GADD45A	-1.08	0.92	0.9922	ns
GAST	-1.32	0.76	0.6517	ns
GDF15	-**4.22**	**0.24**	**0.0327**	*
GPX3	1.8	1.8	0.1291	ns
HBEGF	-1.27	0.79	0.9164	ns
HOXA10	1.81	1.81	0.1918	ns
HOXA11	-1.1	0.91	0.7422	ns
ICAM1	-**2.07**	**0.48**	**0.0404**	*
IGF1	1.24	1.24	0.5136	ns
IGFBP1	-2.44	0.41	0.301	ns
IL11	2.08	2.08	0.1221	ns
IL15	-1.23	0.82	0.2788	ns
IL1A	-1.29	0.78	0.4451	ns
IL1B	-1.66	0.6	0.2715	ns
IL1R1	1.29	1.29	0.6692	ns
IL6	-3.14	0.32	0.195	ns
ITGA4	-1.46	0.69	0.6359	ns
ITGAV	-1.41	0.71	0.2241	ns
ITGB3	6.4	6.4	0.451	ns
KDR	-**6.99**	**0.14**	**0.0115**	*
LAMC2	-1.69	0.59	0.3188	ns
LEP	-1.12	0.9	0.5819	ns
LIF	-1.59	0.63	0.0571	ns
LIFR	1.33	1.33	0.4115	ns
MAOA	-1.73	0.58	0.5141	ns
MID1	1.1	1.1	0.467	ns
MKI67	1.6	1.6	0.2392	ns
MMP2	-1.65	0.61	0.8731	ns
MMP7	-19.16	0.05	0.0729	ns
MMP9	-2.37	0.42	0.2073	ns
MSX1	-1.22	0.82	0.5109	ns
MUC1	**1.85**	**1.85**	**0.0189**	*
OLFM1	-1.25	0.8	0.3683	ns
PAEP	-2.34	0.43	0.1269	ns
PCNA	1.08	1.08	0.3423	ns
PGF	-1.87	0.53	0.1015	ns
PGR	1.27	1.27	0.3233	ns
PRL	1.73	1.73	0.375	ns
PTGS1	-2.12	0.47	0.9528	ns
PTGS2	-1.66	0.6	0.2325	ns
SELL	**2.24**	**2.24**	**0.0454**	*
SFRP4	**2.53**	**2.53**	**0.014**	*
SOD1	-**1.26**	**0.79**	**0.0084**	**
SPP1	-**12.62**	**0.08**	**0.0185**	*
STAT3	1.08	1.08	0.4506	ns
STMN1	**1.57**	**1.57**	**0.013**	*
TGFB1	1.36	1.36	0.177	ns
TIMP1	**1.91**	**1.91**	**0.0363**	*
TNF	1.61	1.61	0.4738	ns
TNFRSF10B	-**1.65**	**0.61**	**0.0033**	**
TP53	1.01	1.01	0.9896	ns
VCAM1	-1.68	0.59	0.2364	ns
VEGFA	-1.47	0.68	0.1865	ns
WNT2	1.86	1.86	0.0508	ns

Data are calculated as both, fold regulation and fold change of irisin treatment *vs.* control. Statistically significant data are presented in bold. ns, non-significant data (p > 0.05); *, p ≤ 0.05; **, p ≤ 0.01; ***, p ≤ 0.001; ****, p ≤ 0.0001.

**Table 4:** Effect of leptin on the expression of genes related to female infertility.

Gene Symbol	Fold Regulation	Fold Change	p-Value	Significance
ANXA2	1.01	1.01	0.9824	ns
APOD	1.55	1.55	0.8023	ns
AR	1.09	1.09	0.5206	ns
AREG	2.55	2.55	0.1545	ns
BAX	-1.08	0.92	0.6137	ns
BCL2	1.09	1.09	0.8415	ns
C2	-1.22	0.82	0.8023	ns
C3	-2.14	0.47	0.2792	ns
CALCA	1.3	1.3	0.9629	ns
CASP3	-1.09	0.92	0.6023	ns
CCL5	-1.78	0.56	0.8546	ns
CCNB1	1.04	1.04	0.8628	ns
CD55	1.12	1.12	0.2053	ns
CDH1	1.43	1.43	0.5925	ns
CFD	1.09	1.09	0.5935	ns
CLDN4	-1.4	0.71	0.2672	ns
COMP	-1.08	0.93	0.9617	ns
CRABP2	-1.17	0.86	0.49	ns
CSF1	-1.81	0.55	0.1526	ns
CTNNB1	1.15	1.15	0.3522	ns
CXCL12	1.02	1.02	0.9724	ns
DKK1	-1.55	0.65	0.1118	ns
EGF	1.26	1.26	0.5105	ns
EGFR	-1.21	0.83	0.33	ns
ESR1	-1.11	0.9	0.5697	ns
ESR2	1.39	1.39	0.7212	ns
F3	1.15	1.15	0.5501	ns
FBN1	1.22	1.22	0.2301	ns
FN1	1.6	1.6	0.0569	ns
GADD45A	1.15	1.15	0.1484	ns
GAST	1.03	1.03	0.8392	ns
GDF15	-2.52	0.4	0.2892	ns
GPX3	1.46	1.46	0.106	ns
HBEGF	1.51	1.51	0.0813	ns
HOXA10	1.76	1.76	0.3294	ns
HOXA11	-1.04	0.96	0.8184	ns
ICAM1	-1.89	0.53	0.1567	ns
IGF1	1.07	1.07	0.8605	ns
IGFBP1	1.31	1.31	0.2512	ns
IL11	1.44	1.44	0.3379	ns
IL15	-1.2	0.83	0.3587	ns
IL1A	-1.23	0.81	0.6621	ns
IL1B	-2.23	0.45	0.1299	ns
IL1R1	1.59	1.59	0.2971	ns
IL6	-1.97	0.51	0.2302	ns
ITGA4	-1.32	0.76	0.0962	ns
ITGAV	-1.07	0.93	0.3291	ns
ITGB3	4.51	4.51	0.9383	ns
KDR	-2.02	0.5	0.3483	ns
LAMC2	-3.23	0.31	0.0598	ns
LEP	-1.46	0.69	0.1327	ns
LIF	-1.1	0.91	0.655	ns
LIFR	1.49	1.49	0.187	ns
MAOA	-1.1	0.91	0.5909	ns
MID1	-1.15	0.87	0.3192	ns
MKI67	1.47	1.47	0.196	ns
MMP2	-1.88	0.53	0.3988	ns
MMP7	-7.72	0.13	0.251	ns
MMP9	-1.56	0.64	0.553	ns
MSX1	-1.08	0.92	0.6698	ns
MUC1	1.16	1.16	0.1898	ns
OLFM1	-1.45	0.69	0.1941	ns
PAEP	-1.49	0.67	0.933	ns
PCNA	1.02	1.02	0.7715	ns
PGF	-1.23	0.81	0.8469	ns
PGR	1.6	1.6	0.0639	ns
PRL	1.39	1.39	0.7569	ns
PTGS1	-1.94	0.52	0.2358	ns
PTGS2	-2.12	0.47	0.1376	ns
SELL	1.31	1.31	0.4546	ns
SFRP4	1.21	1.21	0.6447	ns
SOD1	-1.01	0.99	0.9654	ns
SPP1	-5.29	0.19	0.0955	ns
STAT3	1.02	1.02	0.8683	ns
STMN1	1.26	1.26	0.1182	ns
TGFB1	1.21	1.21	0.4503	ns
TIMP1	1.44	1.44	0.052	ns
TNF	1.82	1.82	0.3258	ns
TNFRSF10B	-1.18	0.85	0.3565	ns
TP53	-1.01	0.99	0.8372	ns
VCAM1	-1.18	0.85	0.762	ns
VEGFA	-1.14	0.87	0.563	ns
WNT2	1.04	1.04	0.6854	ns

Data are calculated as both, fold regulation and fold change of leptin treatment *vs.* control.

## 2 Experimental Design, Materials and Methods

### 2.1 Cells

Human ovarian granulosa cells were purified from follicular fluid samples from patients undergoing *in vitro* fertilization (IVF) procedure by using Percoll PLUS reagent (GE Healthcare, Cat. # 17-5445-02). Initially, follicular fluid was centrifuged at 1000 g for 5 min. Pellet containing the cells was resuspended in 20 mL of PBS and layered onto 15 mL of 50% Percoll PLUS reagent followed by centrifugation at 400 g for 30 min. Granulosa cells were collected from the intermediate layer, washed two times with PBS and seeded in cell culture dishes with DMEM/F12 (50:50) medium (Corning, Cat. # 10-092-CM) supplemented with 10% FBS (VWR, Cat. # 89510-186) and antibiotic/antimycotic mixture (MP Biomedicals, Cat. # 1674049). For experiments, 0.3 × 10^6^ cells were plated in 6-well plates and one day later the cell culture medium was replaced with serum-free medium supplemented with 500 ng/ml of irisin (Enzo, Cat. # ADI-908-307-0010) or 100 ng/ml of leptin (BioVision, Cat. # 4366-02) for 24 h.

#### 2.1.1 RNA extraction and qRT-PCR array

After completing the experiment, cells were washed two times with PBS (Corning, Cat. # 46-013-CM) and total RNA was extracted using TRIzol reagent (Ambion, 15596018). RNA was quantified using NanoDrop One spectrophotometer (Thermo Fisher Scientific) and samples were normalized to 1 µg total RNA, then converted to cDNA using qScript cDNA SuperMix (Quantabio, Cat. # 95048). PCR array was performed using RT^2^ Profiler PCR Array – Human Female Infertility (Qiagen, Cat. # 330231, GeneGlobe ID PAHS-164Z) and QuantStudio 3 Real-Time PCR System (Thermo Fisher Scientific) following manufacturer's protocol.

#### 2.1.2 Data calculation and statistical analysis

Obtained threshold cycle (Ct) values from the PCR array were analyzed using GeneGlobe Data Analysis Center (https://geneglobe.qiagen.com/us/analyze). Gene expression values were normalized using arithmetic mean of the Ct values and the following housekeeping genes: ACTB, B2M, GAPDH, HPRT1, POLP0. Data were accepted as statistically significant if *p* ≤ 0.05.

## Ethics Statements

This research was approved by the Institutional Review Board of Northwell Health (IRB # 20-0449). Informed consent was obtained from all individuals involved in the study.

## CRediT authorship contribution statement

**Radoslav Stojchevski:** Methodology, Validation, Formal analysis, Investigation, Writing – original draft, Writing – review & editing, Visualization. **Tomer Singer:** . **Karina Ziskovich:** . **Leonid Poretsky:** Conceptualization, Writing – review & editing, Supervision, Project administration, Funding acquisition. **Dimiter Avtanski:** Conceptualization, Methodology, Validation, Formal analysis, Resources, Writing – original draft, Writing – review & editing, Visualization, Supervision, Funding acquisition.

## Declaration of Competing Interest

The authors declare that they have no known competing financial interests or personal relationships that could have appeared to influence the work reported in this paper.
